# No right is absolute: the need for a more responsible use of social media

**DOI:** 10.3389/fsoc.2025.1704934

**Published:** 2025-11-28

**Authors:** P. Sreeja Gangadharan, S. P. K. Jena

**Affiliations:** 1 Department of Psychological Sciences, CHRIST Deemed to be University, Bangalore, India; 2 Psychology, Delhi University, South Campus, New Delhi, India

**Keywords:** digital media, human rights, privacy, data protection, ethics, freedom of speech and expression

## Introduction

The online social networks (OSNs) have significantly altered the mode of social communication and information exchange, allowing the users to express themselves instantly and interact with others (Liu et al., 2022). Studies have increasingly shown that: OSN users are ubiquitously unaware of how such acts of sharing images can both directly and indirectly compromise privacy and can lead to unwitting violations of individual privacy for themselves and for others (Nyoni and Velempini, 2018). This threats to privacy has only intensified with the advancement of machine learning, since these tools are often used to easily recognize or edit sensitive visual content of an image to create more visual experience and entertainment. Such personal attacks on digital media platforms has became increasingly illegal, unethical and immoral with the advent of machine learning tools and models.

Sharing images from our various personal and social occasions in life on online social networks (OSNs) has become an indispensable part of our social activities. But many of these images and videos we share casually and playfully at times are serious intrusions in to the personal life of others and this has also increased the risk to privacy invasion. Such irresponsible use of social media have emerged as a significant societal harms causing various issues like misinformation, polarization, mental health concerns, and privacy infringements (Braghieri et al., 2022). Thus in a virtual world what constitute individual responsibility is indeed a value judgement and it is framed around moral and legal obligations. Though responsibility has legal, moral, and social aspects (Birsch, 2004); on infringement of privacy of others in the context of social media usage, only the legal responsibility makes it obligatory for an individual to respond to the system. So if the social media users voluntarily contribute to harmful actions within their control, this legal responsibility makes them liable. But among the millions of users of social media, how many are aware of these legal responsibility?

## Legal frameworks

In the context of internet and digital platforms, we largely discusses about the corporate responsibility and responsibilities of the governments to safeguard the personal data and privacy of it's nationals ignoring the user's duty of care to, adhere to platform policies and laws. The user agreements on all these social media platforms like Twitter (now X) and Facebook (Meta) do include clauses that address legal responsibility for sharing third-party content to restrict its users from hate speech and to ensure one's interactions do not incite violence or spread harmful misinformation. Facebook/Meta has created “Community Standards” that define rules for appropriate conduct and makes the users accountable (Tyler et al., 2021). Thus user agreements insists you to be legally responsible while re-sharing or reposting contents in social medias.

Even though the social media is ubiquitous and habitual and around 4.76 billion people (59.4%) around the global uses these platforms for social networking (Petrosyan, 2023), there are serious ethical concerns about privacy within social media platforms (Elias, 2022). Majority of these users fail to read the policies on user agreements and give informed consent before authorizing the agreement. The extensive length, forced or limited options and over-complexity of these privacy policies often leads to uninformed consent; as when users better understand the notion of privacy this has a financial impact for organizations (Hanlon and Jones, 2023). Thus the corporates may not be interested in addressing the issue of the “uninformed consent” on user agreements. Moreover the age of digital consent is 13 years in United States and both Meta and Twitter allow those aged 13 to join its platforms. But the capacity of a 13 year old as an autonomous agent in understanding their actions and comprehend their consent action is highly questionable (Hanlon and Jones, 2023; Faden and Beauchamp, 1986).

## Challenges

Digital rights include human rights: specifically privacy and data protection in the digital spaces. The Universal Declaration of Human Rights (1948) equally emphasis on Freedom of Speech (Article 19) which promotes right to express opinions publicly on any platforms and also Right to Privacy (Article 12) which controls unwanted intrusion to personal information. When there is a clash between these two, it creates a complex legal, ethical, and societal dilemma (United Nations, 1948).

This is exactly what we have witnessed in the Coldplay concert. Recently, the British rock band, Coldplay has got into such an allegation when they engaged in an unmindful act of filming the personal moments of two individuals who attended their concert, which was later aired in social media and has got wide public attention due to the social status of the individuals involved. In this case, what began as a casual-playful act by Coldplay was amplified and celebrated by the mainstream and social media. This has resulted in mental trauma and humiliation for both the families. Here, the entire sequence of events, combined with overwhelming public scrutiny, must be emotionally exhausting for both the families involved. This act has to be condemned for the unrecoverable damage this has done to their families, their personal and professional life.

Though we cry out loud for our privacy and condemn any intrusion into our personal space and freedom—even by the state for security purposes let alone by others; social media users are often involved in online shaming, public humiliation of others by invading into their personal and private affairs in digital spaces with no remorse. These acts has to be examined for its legal consequences and wide public awareness has to be conducted to avoid such intrusion in to the personal spaces and privacy of others using the digital media platforms in the future.

Talking about the virtual world, we do not carry any moral obligations here and this is a serious issue which requires attention. Studies on what people choose to do in the virtual world are suggested to shed more light on what people will do in a world where they can act without fear of moral sanction due to “virtual anonymity.” Anonymity gives a lot of power to us: power to engage in serious crimes, power to exploit, power to manipulate others through fake stories and framing others for personal gains. This conduct in the virtual world indeed reminds us the traditional challenge to human morality posed by Plato and the magical ring which Glaucon asks us to wear that makes us invisible and capable of acting anonymously (Plato, 2000). As it is rightly said, people are moral only because of the costs to them for being immoral. These actions confirm Nietzsche's metaphorical proclamation: “God is dead. God remains dead. And we have killed him”—a statement about the declining traditional values and beliefs, resulting in a moral and cultural crisis. The human race needs to be warned against the consequences and we should further sharpen our moral compass, and should act with utmost integrity.

Social media is a very dangerous tool that can cause serious destructions in personal life and the society due to disinformation, cyberbullying, and psychological distress (Braghieri et al., 2022; Roth et al., 2024) if not used responsibly. The unprecedented scale and speed of social medias (Ollier-Malaterre and Foucreault, 2021) and the human inclination toward selfishness, underscore the importance of enforceable moral responsibilities, whether through legal systems or social contracts (Sapontzis, 1981). Though this conventional view of punishment for violations is often rooted in a deficit-based approach and aligns with ethical theories such as deontology (Dubljević et al., 2022; Gal et al., 2022) the diversity of human nature and the complexities associated with that limits us from adopting “Virtue ethics,” which centers on individual as the focal agent of moral conduct and emphasizes the significance of personal characteristics in shaping moral actions (Gal et al., 2022). Since the responsible use in this technological contexts are closely tied to direct individual actions, and human by nature has inclination toward selfishness, social media platforms requires robust legal provisions which mandates more effective use which do not infringe on the right and privacy of others.

There exists substantial limitations in addressing the complex issue of digital ethics comprehensively in the research landscape. The gap is not only in the research but also in practice due to the limitations in both the national and International policies. In a recent study which analyzed 71 policy documents from 20 countries and four international governmental organizations (IGOs) comments: distribution of digital ethics topics reflects a broader dynamic between international influence and national adaptation. This underscore the importance of multilevel governance approaches that can provide coherent standards while accommodating local contexts (Guenduez et al., 2025). Hence the combined efforts of governments and private actors in establishing ethical standards with national adaptation and ensuring compliance is the way forward.

## Conclusion

The power of digital media and the social networking sites are immense since it allows anyone to share their views and express themselves without any discrimination, however we also experience considerable difficulties in dealing with the modern privacy issues in such platforms. A strong legal framework, integrating novel privacy analysis with computer-aided privacy-enhancing technologies as privacy intelligence is indeed the need of the hour. This will ensure the contents shared in such online social networking sites do not intrude into the privacy of others.
